# Prognostic factors of oesophageal squamous cell carcinoma from the perspective of molecular biology

**DOI:** 10.1038/sj.bjc.6990499

**Published:** 1999-06

**Authors:** Y Shimada, M Imamura, G Watanabe, S Uchida, H Harada, T Makino, M Kano

**Affiliations:** Department of Surgery and Surgical Basic Science, Graduate School of Medicine, Kyoto University, Shogoin Sakyo-ku, Kyoto, 606-8507, Japan

**Keywords:** oesophageal cancer, molecular marker, biological marker, cyclin D1, E-cadherin

## Abstract

Recent developments in molecular biology have revealed that several oncogenes, suppressor genes and adhesion molecules are involved in the development of oesophageal cancer; however, the role of these genes is still unknown. To evaluate which molecular biological factors are related to patients' prognosis and recurrence, we checked p53, p16, p21/Waf1, cyclin D1, Ki-67, epidermal growth factor receptor (EGFR), vascular endothelial growth factor (VEGF), Mdm2, Bcl2, E-cadherin and MRP1/CD9 by means of immunohistochemical analysis in 116 cases of oesophageal cancer (R0). We also checked the regrowth capability of the primary cultures of the resected tumours and the effect of post-operative treatment. Although univariate analysis revealed that pN (pTNM), pT (pTNM), sex, cyclin D1, Ki-67, VEGF, E-cadherin and cell regrowth capability were prognostic factors, multivariate analysis revealed that pN (risk ratio (RR) 3.17), sex (RR 8.13), cell regrowth capability (RR 3.03) and E-cadherin (RR 0.30) were prognostic factors. Interestingly, step-wise analysis revealed that the following five factors were prognostic factors: pN (RR 5.74), sex (RR 3.14), cyclin D1 (RR 2.29), E-cadherin (RR 0.26) and cell regrowth capability (RR 1.94). Logistic regression analysis revealed that the risk factors of haematogenous recurrence were pN (odds ratio (OR) 8.97), cyclin D1 (OR 4.52) and EGFR (OR 0.18). On the other hand, the risk factor of lymph node recurrence was pN (OR 5.16). With regard to the effect of post-operative treatment, post-operative radiotherapy was a favourable risk factor (RR 0.43) and reduced the haematogenous recurrence (OR 0.18). Our data indicate that combination analysis using pN, sex, cyclin D1, E-cadherin, EGFR and cell regrowth capability may be useful for the prediction of patient survival and recurrence. © 1999 Cancer Research Campaign

